# Scientific support to plant breeding and seed production in Siberia in the XXI century

**DOI:** 10.18699/VJ21.050

**Published:** 2021-07

**Authors:** N.P. Goncharov

**Affiliations:** Institute of Cytology and Genetics of the Siberian Branch of the Russian Academy of Sciences, Novosibirsk, Russia

**Keywords:** breeding, Siberian Federal District, traditional and modern methods, next-generation cultivars, seed production, селекция, Сибирский федеральный округ, традиционные и современные методы, сорта нового поколения, семеноводствo

## Abstract

Agriculture in the Russian Federation is fundamental to the country’s economic performance, living standards, the wellbeing of people and state safety. Considerations relating to food security, prospects of and challenges before plant breeding in the Siberian Federal District (SFD), the largest agricultural area of the Russian Federation, are provided in the article. The agricultural area used in the SFD is about 50 million hectares and accounts for 13 % of the country’s gross grain production. The need for the introduction of modern molecular biological methods, bioengineering and IT technology is demonstrated and discussed. As Russia as a whole, Siberia is largely engaged in unpromising extensive farming practices, which rely on natural soil fertility, and this factor should be taken into account. Another issue is noncompliance with intensive farming technologies used for cultivating new-generation commercial cultivars. Although capital investments in plant breeding are the most cost effective investments in crop production, breeders’ efforts remain underfunded. The article explains the need for fundamental reform in this economic sector: the recognition of plant breeding as being a fundamental science; a fair increase in its funding; the development of a breeding strategy, nationally and regionally; the further expansion of the network of the Breeding Centers; the re-establishment and improvement of the universities’ departments specialized in plant breeding and seed production; having more state-funded places in the universities for training plant breeders to be able to maintain and cement the country’s advanced position in plant breeding and to develop new globally competitive next-generation cultivars of main crops. Should these issues be ignored, all the problems that have accumulated to date will lead to risks of long-term instability in this economic sector. The need for the careful preservation of continuity in plant breeders and plants being bred is stated. The regulatory functions of the state and agricultural science in plant breeding, plant industry and seed production are considered.

## Abstract (ru)

Аграрное производство Российской Федерации представляет собой стержень экономической системы государства, от развития которого во многом зависит уровень жизни и благосостояния населения. Оно обеспечивает основу безопасности страны. В статье рассматриваются вопросы продовольственной безопасности Сибири, перспективы и проблемы селекции растений в Сибирском федеральном округе – самой крупной аграрной территории РФ. Площадь сельскохозяйственных угодий в Сибирском федеральном округе около 50 млн га, здесь производится 13 % валового объема зерна страны. В Сибири, как и во всей России, преобладает бесперспективное экстенсивное земледелие, основанное на эксплуатации естественного плодородия почв, поэтому селекция должна учитывать не только несоблюдение технологий возделывания новых сортов интенсивного типа, но и этот немаловажный фактор. Несмотря на то что капитальные вложения в селекцию являются наиболее окупаемыми в растениеводстве, селекционеры до сих пор работают в сложных экономических условиях. Обсуждается использование в селекции современных молекулярно-биологических методов, биоинженерии и IT технологий. Обосновывается необходимость проведения кардинальной реформы отрасли, включая признание селекции фундаментальной наукой, существенное увеличение ее бюджетного финансирования, определение стратегии ее развития на федеральном и региональном уровнях, дальнейшее целенаправленное расширение сети селекционных центров, восстановление и укрепление в аграрных университетах профильных для отрасли кафедр селекции и семеноводства, выделение бюджетных мест в вузах региона для подготовки селекционеров, способных сохранить и упрочить передовые позиции страны в селекции растений и создавать конкурентоспособные на мировом аграрном рынке сорта нового поколения основных сельскохозяйственных культур. В противном случае накопившиеся к настоящему времени проблемы могут привести к возникновению новых долгосрочных рисков нестабильности в отрасли. Делается вывод о необходимости бережного сохранения преемственности, как специалистов, так и селекционного (селектируемого) материала. Рассматриваются особенности регулирующих функций государства и аграрной науки в селекции растений и в отрасли в целом.

## Introduction

The food sovereignty of the Russian Federation is the basis of the country’s safety. This strategically important point is documented in normative acts. Challenges of the 21st century in Russia’s and its Siberian Federal District’s agricultural sector are well known: global and local climate changes (Gurova, Osipova, 2018) entailing the drying out of soils in the southern areas, which are most favorable for farming, a continued decline in soil fertility (Syso, 2017) and a reduction in the working rural population (Shabanov et al., 2019). All these factors lead to new long-term risks to the stability of the agricultural market. Recently, the concept of smart agriculture has become more and more popular worldwide (Anischenko, 2019). In the Russia of today, it means that agriculture is supposed to proceed on its own. This is a good old national tradition: the Ministry of State Property of the Russian Empire, together with the Department of Agriculture as its part, did not seem to think too much about how scientific achievements could be applied to agriculture, nor did they seem to think about agriculture itself (Elina, 1995, p. 45). According to Elina, that negligence stemmed from a popular belief in the Russia of the 19th century that farming was Russian peasants’ natural occupation, and so it was supposed to evolve by itself, without governmental or scientific involvement (p. 45).

The agricultural area used in the Siberian Federal District (SFD) is about 50 million hectares and accounts, according to the Federal State Statistics Service (rosstat.gov.ru), for 13 % of the country’s gross grain production. Before 2013, scientific support to regional agriculture was provided by the Siberian Branch of the Russian Academy of Agricultural Sciences. The regional responsibility for the development of agricultural science has since been delegated by the Department of Agricultural Sciences of the Russian Academy of Sciences and the Ministry of Science and Higher Education of the Russian Federation to the Siberian Federal Scientific Center for Agrobiotechnology (Krasnoobsk, Novosibirsk region) created out of the former Presidium of the Siberian Branch of the Russian Academy of Agricultural Sciences and several agricultural research institutes in Novosibirsk region, Tomsk region, Kemerovo region and Zabaykalsky Krai, to the Omsk Agrarian Scientific Center and to the Federal Altai Scientific Center for Agrobiotechnology (Barnaul). With that done, the entire agrarian area of Eastern Siberia and the Extreme North has virtually been left without scientific support. The Joint Academic Council of Agricultural Sciences of the Siberian Branch of the Russian Academy of Sciences (with Full Member of the Russian Academy of Sciences N.I. Kashevarov as Chair and Prof. I.M. Gorobey as Academic Secretary) is the only entity that is doing its best to keep running scientific support. The Joint Academic Council has the Joint Academic and Topical Council of Plant Industry, Breeding, Biotechnology and Seed Production (now with Full Member of the Russian Academy of Sciences N.P. Goncharov as Chair), which was created in 1972 at the Presidium of the Siberian Branch of the V.I. Lenin All-Union Academy of Agricultural Sciences and has since 1992 been operating at the Presidium of the Siberian Branch of the Russian Academy of Agricultural Sciences. The Council provided scientific and methodological guidance to the Siberian Breeding Centers and coordinated their cooperation (Shumny et al., 2016). Before the COVID-19 pandemic, the Council had held 46 annual retreat sessions, with the last event having taken place in Krasnoyarsk, July 23–26, 2019^1^. The sessions were traditionally timed to coincide with plant breeding conferences: originally with national only and later with national and international; and the participating organizations were supposed to display their newest commercial regional cultivars for comparison. The venues for the sessions alternated between different Siberian agricultural plant production institutions.

^1^ Theses of the International Scientific Conference “Optimization of the Breeding Process – a Factor of Stabilization and Growth of Crop Production in Siberia” OBP–2019. Krasnoyarsk, 2019.


Before ‘reorganized’ (eliminated) in 2013 and losing its institutes to the Federal Agency for Scientific Organizations of the Russian Federation, the Russian Academy of Agricultural Sciences had had eight specialized Breeding Centers in the Siberian Federal District:

- Siberian Research Institute of Agriculture (B.I. Gerasenkov, K.G. Aziev, R.I. Rutz)^2^;
- Altai Research Institute of Agriculture (V.I. Kandaurov, V.I. Yanchenko, N.I. Korobeinikov);
- Krasnoyarsk Research Institute of Agriculture (N.A. Surin);
- Siberian Research Institute of Plant Production and Breeding (P.L. Goncharov, I.E. Likhenko);
- Siberian Research Institute of Fodder Crops (A.V.Zheleznov, R.I. Polyudina);
- Lisavenko Research Institute of Horticulture for Siberia (I.P. Kalinina, I.A. Puchkin, T.N. Nelyubova);
- Research Institute of Agriculture of Northern TransUrals (V.V. Novokhatin);
- Kemerovo Research Institute of Agriculture (V.N. Pakul’).

^2^Heads of the Breeding Centers appear in parentheses in chronological order


All these institutes, except the Altai Research Institute of Agriculture (now the Federal Altai Scientific Center for Agrobiotechnology) and the Siberian Research Institute of Agriculture (now the Omsk Agrarian Scientific Center), are now the branches of other organizations.

These Breeding Centers worked according to two 20-year programs: 1971–1990 and 1991–2010 (Program…, 1978, 1989). A third 20-year program, 2011– 2030, has been elaborated for each of these eight breeding centers (see, for example, Program…, 2011a, b). In addition, there were Siberian regional scientific programs (‘Diallel Analysis’ (Dragavtsev et al., 1984) and ‘Siberian Wheat’ (Goncharov P.L. et al., 1989)) as well as All-Union and All-Russian goal-oriented programs (Goncharov N.P., Shumny, 2006), including the AllUnion program “Lucerne” (Goncharov P.L., 2009). Information on cultivars zoned for Siberia is provided in a systematized manner in a four-volume set, Catalogues… (see, for example, Catalog…, 2009)

In 2020–2021, the Ministry of Science and Higher Education of the Russian Federation organized 36 narrowly specialized Breeding and Seed Production Centers, of which only four are in Siberia: three in Western Siberia (Omsk region, Kemerovo region and Altai Krai) and one in Eastern Siberia (Krasnoyarsk Krai). Of note, this was the third attempt to organize Breeding Centers in the country. Of the two previous attempts, one had been made by Nikolai I. Vavilov in 1929 (Goncharov N.P., 2017) and one, by A.V. Pukhalsky in 1972 (Shumny, Goncharov P.L., 2008)

Although investments in breeding are the most rewarding in crop production, breeders are still struggling:

- they are permanently short of material and human resources;
- the system that used to ensure seed production and the introduction of new commercial cultivars has now been destroyed;
- the sector has been suffering chronic underfunding ever since Mikhail Gorbachev’s rule. As a result, there is a continuous search for funding to ensure the country’s food security.

**Fig. 1.**
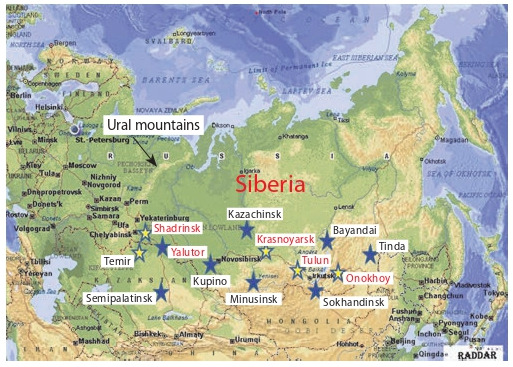
The network of research agrarian institutions in Siberia at the beginning of the 20th century. Experimental Fields were organized in 1907 in Tulun and Temir, in 1908–1910 in Yalutor, Shadrinsk, Kupino, Krasnoyarsk, Minusinsk, Kazachinsk, Bayandai, and Onokhoy and in 1911 in Semipalatinsk and in other places.

At the moment, the amount of scientific patronage over crop production in Siberia is short of that taken at the beginning of the last century (Fig. 1). In the Siberian Federal District, breeding activity has been discontinued for many crops for the lack of high-quality human resources. In Eastern Siberia, only two regional agricultural research institutes, branches of the Krasnoyarsk and Irkutsk Scientific Centers of the Siberian Branch of the Russian Academy of Sciences, are still active in plant breeding. Agrarian science has always been much less developed and much less sustainable in Siberia than in the European part of the country.

Shortly after elimination of the Russian Academy of Agricultural Sciences and “withdrawal” of the responsibility for scientific support to agricultural science in the Siberian Federal District from its former institutes, the annual release of recommendations for field work was discontinued (Alkhimenko et al., 2015; Donchenko et al., 2015), as they do not count as performance indicators as per the Ministry of Science and Higher Education of the Russian Federation. Stable yield and grain harvest in the SFD is now the responsibility of this ministry

## Pre-breeding

Soils. Although soils represent the most important resource for humankind, the attitude towards them in Siberia and throughout the Russian Federation is not consistent with their importance for ensuring the country’s food security (Chekmarev, 2018). Companies and farmers seek to secure their right to land through state registration; however, they are unaware of the fertility status of the soils to become theirs, nor are they responsible for maintaining and enhancing soil fertility (Rassypnov, Ushakova, 2017). Additionally, neither the powers that be nor the Ministry of Science and Higher Education of the Russian Federation recognize the experimental fields of the research institutes as being a unique expensive research tool.

Nowadays, the depletion of soil fertility largely determines possible uses for the soils in the future and the vectors of development of breeding and seed production. Soils have been “watered with our tears and fertilized with our inactivity” for more than three decades. Farms and joint-stock companies in the region have shifted to an extensive three-field crop rotation system everywhere. It should be noted that some modern technologies, such as no-till cultivation, interfere with the process of mineralization of organic matter, which leads to a decrease in the accumulation of nitrogen in the soil (Korchagina, Yushkevich, 2017). Ridge tillage, too, is not universal (Elina, 2017) and does not promote soil fertility in agricultural lands.

**Fig. 2.**
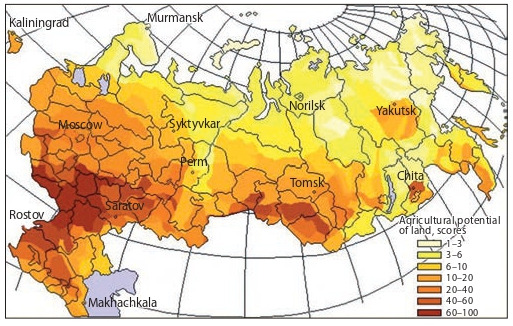
Soil resources of the Russian Federation (agricultural potential of land). URL: https://studfile.net/preview/1758647/page:3 (Acccessed Nov. 10, 2019).

**Fig. 3.**
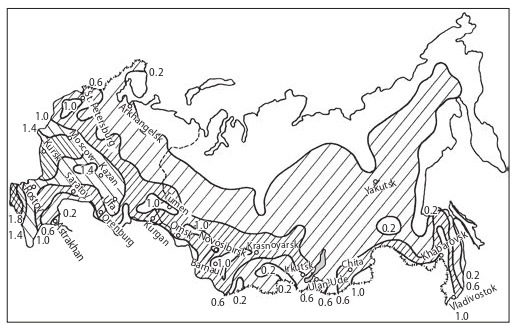
Agro-climatic potential of the Russian Federation (from: Siberia…, 1993). “1” stands for the country-average.

There is an urgent need to expand arable land for the further development of the sector in a number of regions (Renev et al., 2017). At the same time, the problem with increasing crop production in the SFD is being addressed extensively, that is it is unlikely that someone in the Russian Federation, not to say in Siberia, may have the will to invest in agricultural science, looking at the spaces of abandoned farmland. Inferior to the European part of the Russian Federation in terms of soil quality (Fig. 2) and agro-climatic potential (Fig. 3), Siberia can only rely on breeders. Incidentally, the introduction of scientific developments is perhaps the only successful part there, as the other resources for maintaining the existing volume of crop production in Siberia are scarce. It is sad to observe that the SFD authorities are not fully aware of their responsibility before the future generations of Siberians for an uninterrupted food supply and that said authorities allow the Ministry of Science and Higher Education of the Russian Federation to bull-headedly “reorganize” experimental agricultural institutions in Siberia. It should be mentioned that over the past hundred years this has been the country’s first reform in agricultural science with neither local authorities not Ministry of Agriculture involved (Chernoivanov, 2006). Too bad, there are no such entities in the Siberian Federal District as agricultural holdings that would at least keep local seed production running; while the reformers say plant breeding should be competitive at all stages and get quick profits. Came up with a cultivar? Get it sold! At the same time, the state is not a regulator of the agricultural market.

Gone are the days when the average grain yield was substantially higher in Siberia than in the European part of the Russian Empire and the tsarist government had to set up the Chelyabinsk customs with a duty to tax outbound crops so that the Siberian peasant would not flood the rest of Russia with cheap grain (Goncharov N.P., Goncharov P.L., 2018). In response, the Siberian peasants organized themselves in production cooperatives and began to export to Western Europe and the exported products were not grain or flour, but oil and cheese. It is estimated that the production of 1 kg of butter requires 25 liters of 3.5 % milk or 25 kg of grain, because to produce 1 liter milk, about 1 kg of grain is needed. To produce 1 kg of milk powder, 11 liters of milk is needed. To produce 1 kg of meat, 15 kg of grain is needed. On average, 1 ton of class 3 wheat can yield about 600 kg of premium flour and 100 kg of grade 1 flour, with the remaining product being bran. Deep processing can further reduce cargo carriage volumes.

In the near future, crop production in the Siberian Federal District and, therefore, the main breeding priorities will become dependent on a grain terminal to be constructed in the Bay Troitsy (Primorsky Krai), with a total capacity of 10 million tons of grain per year. This link in the logistics chain for exporting from Siberia to the countries of the Asia-Pacific region will further increase the imbalance in the plant production.

Breeding strategy. To develop a good strategy for future breeding process, awareness of the current and past experience should be raised. N.I. Vavilov (1935) noted that breeding is made up of knowledge about the initial material, about variability and heredity, and about the role of the environment; from hybridization theory and plant breeding theory; from knowledge about immunity, resistance to adverse abiotic factors and breeding for quality. Recently, high-tech technologies have become yet another part of it. 

Time taken to develop a new cultivar. A long-term plan for a new cultivar is going through a series of successive stages and is expected to be a solid achievement success on completion. The cultivars that the State Variety Testing System includes in the State Register of Breeding Achievements of the Russian Federation will occupy vast areas not sooner than in the next few years. The best cultivars that the breeders will submit to the State Variety Testing System today will be included in the “State Register…” only three years later and, therefore, will occupy large areas only five or ten years after. The cultivars that are being created today will go into production only after 20 years (it takes them 15 years to be developed and passed competitive or ecological tests, 3 years to be considered by the State Variety Testing System and 3–4 years to occupy substantial areas) (Goncharov N.P., Goncharov P.L., 2018). Thus, before proceeding to developing a new cultivar, the breeder should set strategic objectives and outline ways to achieve them, not forgetting that in 20 years cultivar requirements may become quite different due to possible changes in criteria, economic situation, cultivation and processing technologies.

However, the development duration that long does not represent a problem with any ongoing breeding process, because the variety development “conveyer” keeps running and new cultivars are being permanently submitted to the State Variety Testing System. The problem is only how to ensure an inflow of professionals and breeding material – and that should be the concern of the state and agricultural science.

Cultivar model. Because the development of a cultivar should be thoroughly planned, and the plan should be clearly defined, the model or the idiotype of the cultivar should be identified first. The cultivar model for individual traits (the cultivar idiotype) has existed for a long time (Donald, 1968). The first models were local cultivars cultivated by peasants. It is reasonably believed that breeding for a particular idiotype is largely breeding for elimination of deficiencies (Davies, 1977), that is, a plan for improving one or more characters of the plant being worked on. However, because “critical characters” can be revealed only after the cultivar model has been described, the role of its specification becomes unclear (Kazak, Loginov, 2019).

Expending and conservation biodiversity. Although rich collections of cultivated species have been taken up, most of accessions have lost their former genetic potential. The problem is not that the world VIR collection is currently only the fourth largest collection in the world, but that it has been left unsupplied with the accessions of the best foreign commercial cultivars for the past 30 years. This has already alerted VIR competitors. Thus, the plant gene collections – Siberian (see the Table) and others (Goncharov N.P., Shumny, 2008; Kershengolts et al., 2012; Levitskaya, 2017; Kosolapov et al., 2021) – will sooner or later obtain accreditation and turn into regional and national highly specialized genebanks. To date, dozens of institutions have been included in the National Plant Germplasm System of the USDA. And what we have got on our side? It has taken us 10 years… not to have yet commissioned the Federal Permafrost Seed Repository in Yakutsk (Kershengolts et al., 2012).

**Table 1.**
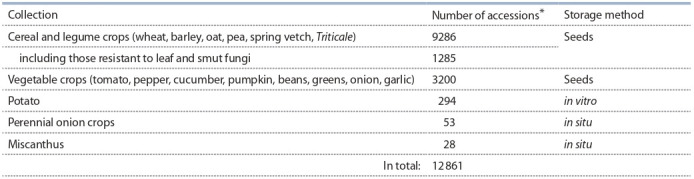
Collections of crops kept as living accessions in the Siberian Research Institute of Plant Production and Breeding, a branch of the Institute of Cytology and Genetics of the Siberian Branch of the Russian Academy of Sciences (Likhenko I.E., p.c.) * There is collection of 387 arboreal taxon.

The problem of conservation and effective use of biodiversity is closely related to plant variety right. Assoon as a cultivar enters the genepools’ collections, it automatically becomes their property (see, for example, the website of VIR).

Another way to deal with the dissipation of “gene bank assets” is through international cooperation. In 2000, CIMMYT people organized the Kazakhstan-Siberia Network on Spring Wheat Improvement (KASIB). The goals are: (1) to screen modern breeding materials (including those provided by CIMMYT) through environmental testing; (2) to increase genotypic diversity and to accelerate the breeding process; (3) to exchange the experience and professional development of breeders of the Russian Federation and the Republic of Kazakhstan, including participation in conferences, seminars and internships in CIMMYT divisions. In addition, a number of foreign breeding and seed production companies have come to Siberia on easy terms. The German breeding company “Strube” is successfully operating in Altai, together with local breeders (Korobeinikov et al., 2020). Admittedly, the Siberian Federal District cannot boast having created subdivisions of KWS or other breeding and seed production giants, which the European part of the Russian Federation can.

## Traditional breeding

The use of traditional methods has already been reviewed in detail (see, for example, Goncharov N.P., Goncharov P.L., 2018). Let us briefly consider the main of them.

Distant hybridization. Uses of distant hybridization in breeding in Siberia were suggested in the 1930s by N.V. Tsitsin and have since given real results. The first winter cultivars of Triticale in the Siberia were produced on the basis of V.E. Pisarev’s amphidiploids (AD), and the world’s first commercial Triticale cultivar ‘Rosner’, on the basis of spring accession ‘AD-20’. Distant hybridization as a breeding method has again become popular in Siberia (Stepochkin et al., 2012). It can be considered an alternative to GMOs, because it allows the transfer of the desired genes between plant species without the use of vectors (genetically engineered constructs).

Polyploid forms. Polyploid cultivars of rye (Likhenko et al., 2014) and clover (Polyudina, 2016) have the highest yield and high level of winter hardiness in Siberia. Unfortunately, triploid beet cultivars and polyploid cultivars of a number of vegetable crops can no longer be found in Siberian fields.

**Fig. 4.**
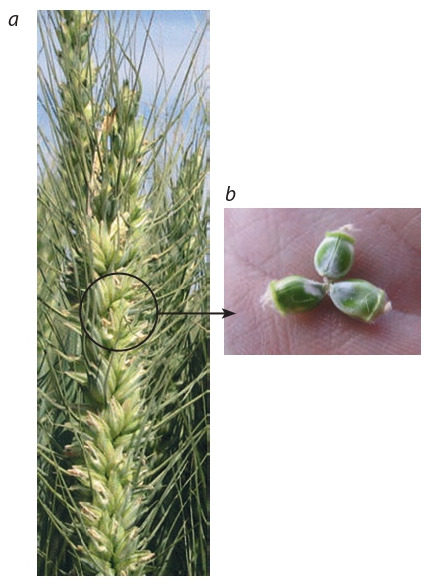
A common wheat mutant with three grains per flower: a, spike; b, grains (from: Manual Book…, 2009). Photo by V.P. Shamanin, Omsk State Agrarian University.

Breeding for productivity and grain quality. The yield of common spring wheat cultivars in Western Siberia depends on the number of plants preserved for harvesting and kernel weight per spike (Kazak, Loginov, 2019). Productive tillering is 1.2 stems per plant. In this case, breeding for the optimization of ear architectonics is possible (Konopatskaia et al., 2016), and so is breeding for branch spike (Dobrovolskaya et al., 2017), for other characters of ear architectonics (Fig. 4) and for close (heavy) seedlings by using mutes with erected leaves (Dresvyannikova et al., 2019), as is the case with some corn cultivars.

Duration of vegetation period. Early maturation is an important breeding character throughout our country (Vavilov, 1935). Most spring wheat varieties in Russia carry two dominant Vrn genes, which control the expression of this character. New alleles of these genes have promise for changing vegetative duration in common wheat by breeding (Chumanova et al., 2020). For most cultures, the genetics of this character is poorly studied, which prevents an extensive use of molecular biological methods.

Disease resistance. A phytopathological assessment of the collection of local and commercial Siberian common spring wheat cultivars showed that only 10–15 % of the accessions had low susceptibility to leaf (brown) and stem rust and powdery mildew (Leonova et al., 2017). To increase the diversity of new genes that control resistance to these pathogens, it is efficient to perform their introgression from wheat relatives (Orlovskaya et al., 2020; Adonina et al., 2021), including artificial amphiploids (Goncharov N.P. et al., 2020). The common spring wheat cultivar Grenada with horizontal resistance to stem rust has been developed as an alternative using of effective resistance genes in breeding by the Research Institute of Agriculture of Northern Trans-Urals – a branch of the Tyumen Scientific Center (Novokhatin et al., 2019).

## Non-conventional breeding

Breeding and improving soil fertility. Crop rotation was a former practice in Siberia to improve soils with, including the obligatory cultivation of legumes and perennial forage grasses (Ilinykh, 2016). Currently, a new direction in breeding has come to the scene: the commercial grain wheatgrass cultivar ‘Sova’ was developed for regenerative agriculture with the accumulation of carbon in the soil (Shamanin et al., 2021). It has been included in the “State Register…” since 2020 as an alternative to perennial wheat. It is simultaneously cultivated for grain, which is harvested before the green mass dies, and for hay. Its grain yield is 9–10 tons per ha/year and hay yield is 7–7.5 tons per ha/year. Sowing is used without replanting for up to 7 years. It is drought- and diseaseresistant. ‘Sova’ is extremely interesting in view of the Russian Federation’s ratification of the Paris climate agreement, because this variety is capable of accumulating up to 3.7 tons per ha carbon annually in the soil.

Functional nutrition is directly related to the longevity of a person. For this reason, it is believed outside Russia that it is cheaper and more profitable to properly feed people than treat them (Fotev et al., 2018). However, this requires that plants be bred for specific nutrients. In Russia, about 80 % of commercial vegetable production accounts for the so-called “borscht set”: white cabbage, tomato, cucumber, carrot, table beet and onion^3^. The assortment can be significantly expanded both by new vegetable crops and new grain crops. For example, it is possible to bake “healthy bread” from wheatgrass (Thinopyrum intermedium (Host) Barkworth & D.R. Dewey) grain, which contains five times as much calcium and 10 times as much folic acid than bread from common wheat (Shamanin et al., 2021). Cereal varieties with increased contents of trace elements can be used (Abugalieva et al., 2021).

^3^ Chekmarev P. A. The state, prospects of development and measures of state support for vegetable growing. URL: https://agrotip.ru/wp-content/uploads/ 2018/11/Presentatsia_Petra_Chekmareva.pdf (Accepted 14.01.2021).


## Molecular biological methods in plant breeding

Breeders have received new tools to improve the genotypes of cultivated plants. The Institute of Cytology and Genetics of the Siberian Branch of the Russian Academy of Sciences took part in the assembly of the wheat genome (IWGSC…, 2018). The next stage of breeding in the future is working with the pan-genome of economically important plants (Pronozin et al., 2021a).

**Fig. 5.**
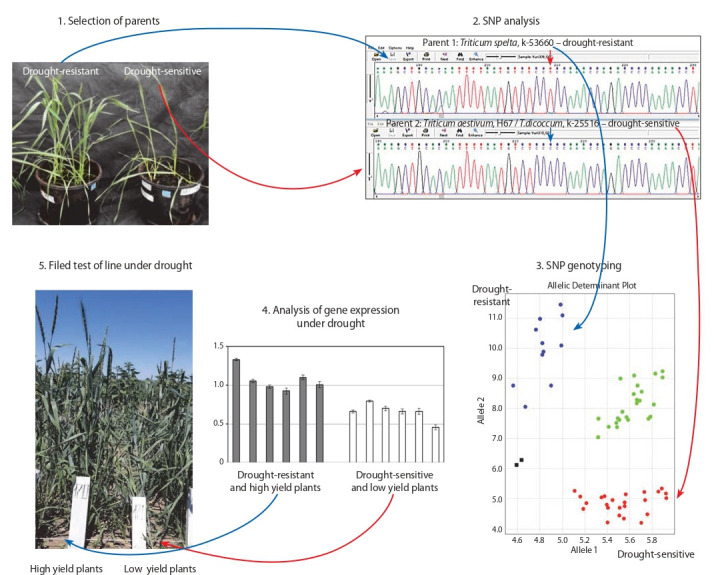
Production of drought-resistant spring wheat cultivars with high yield and grain quality for the southern part of Western Siberia and Northern Kazakhstan using modern molecular biological methods (Jatayev et al., 2020).

Breeding for resistance to adverse abiotic factors. A variant of breeding for drought resistance using methods in molecular biology is shown in Fig. 5.

Plant height and architectonics. Breeding for short stems is once again among the priorities of Siberian breeders (Korobeynikov et al., 2020). Selection for genes that control the optimal plant height can now be effectively carried out by molecular markers (Sukhikh et al., 2021).

Genetic modifications to improve cultivated plants. Breeders widely use genomic modeling and editing technologies to address plant breeding problems (Salina, 2016). Modern systems demonstrate the possibility of obtaining non-transgenic plants with specified mutations (Borisuyk et al., 2019), for example editing the genes that control the optimal flowering time of the most important crops (Kishchenko et al., 2020).

## Bioinformatics

New challenge for bioinformatics is the development of IT, providing a quick, accurate, massive and at the same time detailed description of plant phenotypes both in the field and in laboratory settings (Kolchanov et al., 2017; Genaev et al., 2019). At the same time, it is necessary to substantially reduce the cost and time of obtaining relevant data with the maximum coverage, resolution and dynamics. For the diagnosis of the state of crops, it is promising to use drones (Alt et al., 2019); for the diagnosis of plants, automatic morphology phenotyping (Pronozin et al., 2021b); for a risk assessment of edited plant organisms, dynamic programming (Korotkov et al., 2021).

## Varietal seed production and varietal authenticity control

Cultivar authenticity control.Identification of cultivars grown from seeds delivered by non-Russia’s companies is often not possible due to the lack of standard accessions from originator (Lobach, Samus, 2018); it is only possible to assess how cultivar’s seed dockage has.

Foreign cultivars. Foreign and non-local commercial cultivars of grain crops have been successfully displaced from the Siberian Federal District by local breeding cultivars (Loginov et al., 2016; Surin, 2019). At the same time, the seeds of foreign vegetable cultivars just have no rivals. The same goes to many other crops, for example, leguminous ones (Kazydub et al., 2020): for a list of reasons, their importsubstitution does not occur.

Cultivar change is important for the dynamic development of crop production in the Siberian Federal District. There is no doubt that the so-called “varietal mosaic” helps control diseases in the fields (Bespalova, 2016). At the same time, the cultivar should be cultivated for as long as it can provide a stable, high-quality yield. For example, the potato cultivar Russet Burbank developed at the end of the 19th century, is still grown in the United States and Australia for the production of chips. It should be kept in mind that F1 hybrid varieties substantially distort the statistics of the dynamics of variety substitution in the country

## Conclusion

Agriculture in the Russian Federation is the backbone of the country’s economic system and is fundamental to the living standards and wellbeing of people. This sector sets the pattern of the future development of the Russian state (Belyaev, 2018) and, therefore, addressing its issues, including those with breeding and seed production, should be one of the primary tasks of state authorities. Plant breeding is a lasting process, and so it is extremely important to be sure that we have now chosen the right way to go

To the Government of the Russian Federation. Breeding should be given the status of a fundamental science, as there is nothing more fundamental in the world than feeding people and defeat hunger. Not only does breeding deserve a conspicuous place in the national program “Science”, but it also should be funded from the state budget through dedicated funds. Neither self-financing nor self-supporting should be an option.

Teaching future breeders is an important factor in ensuring the country’s food security. For the breeders to be duly taught, it is required that (1) the breeding and seed production chairs be fully restored in all universities of the Ministry of Agriculture; (2) more state-funded places be allocated to breeder students; (3) the state agricultural universities of the Ministry of Agriculture, which, for a reason unknown, live up to the educational standards set up by the Ministry of Science and Higher Education of the Russian Federation, become relevant

To the RAS Department of Agricultural Sciences and the SFD Ministries of Agriculture. There is an urgent need for the adoption of a regional resolution with a title “On the normalization of genetic-based breeding research and launching original seed production in the Siberian Federal District” – unless we want to live up to Project “Breeding 2.0” by the National Research University Higher School of Economics and the Federal Antimonopoly Service of the Russian Federation.

## Conflict of interest

The authors declare no conflict of interest.
